# Thyroid Dysfunction in Patients with Abnormal Uterine Bleeding in a Tertiary Care Hospital: A Descriptive Cross-sectional Study

**DOI:** 10.31729/jnma.5033

**Published:** 2020-05

**Authors:** Minaxi Thakur, Meenu Maharjan, Heera Tuladhar, Yam Dwa, Sunita Bhandari, Smrity Maskey, Manisha Bajracharya

**Affiliations:** 1 Department of Obstetrics and Gynecology, KIST Medical College, Imadol, Nepal

**Keywords:** *hyperthyroidism*, *hypothyroidism*, *thyroid*, *uterine bleeding*

## Abstract

**Introduction:**

Abnormal uterine bleeding is a common gynecological presentation, accounting for at least 20% of all new outpatient visits. It has been recognized that thyroid dysfunction may have profound effects on the female reproductive system. Both hypothyroidism and hyperthyroidism are associated with a variety of changes, including delayed onset of puberty, anovulatory cycles, and abnormally high fetal wastage. Hence, this study was conducted to know the thyroid status of the patient with abnormal uterine bleeding.

**Methods:**

A descriptive cross-sectional study was conducted in all the patients with abnormal uterine bleeding in a tertiary care hospital from 2 August 2019 to 2 February 2020. Ethical clearance was received from the institutional review committee of KIST Medical College. Convenient sampling was done. Data was collected using a questionnaire which includes patients profile, the pattern of abnormal uterine bleeding, and thyroid profile. Statistical analysis was done using Statistical Package for the Social Sciences version 23.

**Results:**

Out of 79 patients, it was found that 67 (84.8%) were euthyroid, 11 (13.9%) were hypothyroid, and 1 (1.2%) was hyperthyroidism. The most common type of abnormal uterine bleeding was menorrhagia 34 (43%), followed by polymenorrhoea 23 (29%), oligomenorrhoea 13 (16.5%), menometrorrhagia 6 (7.6%), metrorrhagia 2 (2.5%), and hypomenorrhea 1 (1.3%). The maximum number of patients was between 20-25 years with the mean age of 31 years. Among hypothyroid, 7 (8.8%) had subclinical hypothyroidism and 4 (5%) had frank hypothyroidism.

**Conclusions:**

Most females with abnormal uterine bleeding were euthyroid. Menorrhagia was the most common pattern of abnormal uterine bleeding.

## INTRODUCTION

Abnormal uterine bleeding (AUB) is one of the most common clinical presentations.^1^ It occurs in 10-20% of women between 15-50 years of age.^2^ This may significantly affect the quality of life,^3^ results in time off work,^4^ lead to surgical interventions including hysterectomy,^5^ and ultimately have a significant impact on the health care system.^6^ The causes of abnormal uterine bleeding are polyp, adenomyosis, leiomyoma, malignancy and hyperplasia, coagulopathy, ovulatory disorders, endometrial causes, iatrogenic, not classified.^7^

Among them, the ovulatory disorder is the most common which occurs secondary to thyroid dysfunction. Many studies like Danese MD et al. and Douglas L Wilansky et al. any menstrual irregularity in non-pregnant women justifies screening for thyroid disorders.^8-9^ Thus thyroid dysfunction may have profound effects on the female reproductive system. Both hypothyroidism and hyperthyroidism are associated with a variety of changes in reproductive function, including delayed onset of puberty, anovulatory cycles and abnormally high fetal wastage.^10^

The aim of the study is to find the prevalence of thyroid abnormality in abnormal uterine bleeding patients from puberty to those who have not attended menopause.

## METHODS

This is a descriptive cross-sectional study that was carried out in the department of obstetrics and gynecology at KIST Medical College, Imadol, Lalitpur from 2nd August 2019 to 2nd February 2020. Ethical clearance was received from the Institutional Review Committee of KIST Medical College. All patients of abnormal uterine bleeding attending the gynecology department during this period from puberty to those who have not attended menopause were included. Patients under hormonal treatment, using contraceptives, having bleeding disorders and pregnant patients were excluded from the study.

Verbal consent was taken. The convenient sampling method was applied. A complete history was taken from all patients with regards to age, parity, menstrual history, onset and duration of complaints, amount of blood flow and any other specific complaints. Following which a thorough examination was carried out which includes a general physical examination, systemic examination, gynecological, and pelvic examination in married women. All the findings were noted down in a predesigned questionnaire. Basic routine investigations like hemoglobin, platelet, blood test, and ultrasound of abdomen and pelvis were performed. Then the serum triiodothyronine (T3), thyroxine (T4), and thyroid-stimulating hormone (TSH) level were measured in all patients. Patients with thyroid-stimulating hormone <0.5 mIU/ml were considered as hyperthyroid and thyroid-stimulating hormone >5 mIU/ml were considered as hypothyroid. The normal ranges of serum thyroid-stimulating hormone, triiodothyronine, and thyroxine in the hospital laboratory were 0.35 to 5.5 mlU/ml, 2.3 to 4.2 pg/ml, and 0.89 to 1.76 ng/dl.

The sample size was calculated by using formula,

n= Z^2^ × p × q / e^2^

= (1.96)^2^ × (0.5) × (0.5) / (0.11)^2^

= 79.33

= 79

Where,

n= sample size
p= prevalence, 50%
q= 1-p
e = margin of error, 11 %
Z= 1.96 at 95% Confidence Interval

A total of 79 patients were included in our study after satisfying all inclusion and exclusion criteria. The data were collected and entered in MS-Excel 2007 and analyzed using the Statistical Package for Social Sciences (SPSS) version 20 software.

## RESULTS

Out of 79 patients in the study, twelve (15.1%) had thyroid disorders, 67 (84.8%) were euthyroid. Out of 12 patients, eleven (13.9%) had hypothyroidism and 1 (1.2%) had hyperthyroidism. Out of 11 patients, seven (8.8%) had subclinical hypothyroidism and 4 (5%) had frank hypothyroidism (Table 1).

**Table 1:** Thyroid profile of patients.

Thyroid leve	Triiodothyronine (2.3-4.2) n (%	Thyroxine (0.89-1.76) n (%	Thyroidstimulating hormone (0.35-5.5
Normal	71 (89.8)	70 (88.6)	67 (84.8)
Increased	7 (8.8)	6 (7.5)	11 (13.9)
Decreased	1 (1.2)	3 (3.7)	1 (1.2)

**Figure 1:**
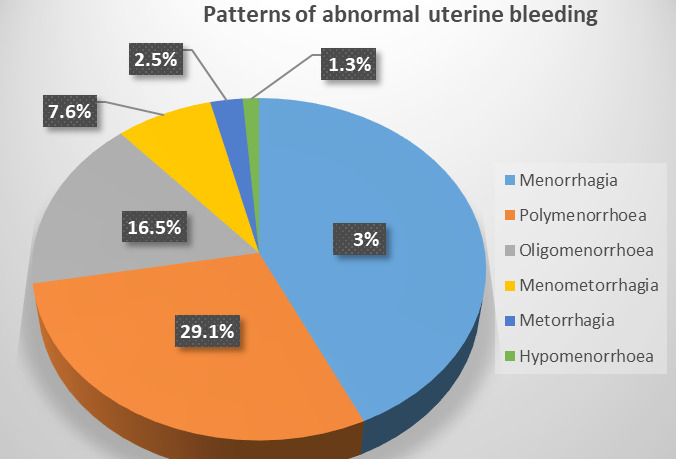
Patterns of abnormal uterine bleeding of patients.

Out of all the types of menstrual irregularities, patients with menorrhagia 34 (43%), polymenorrhoea 23 (29.1%), oligomenorrhoea 13 (16.5%), menometrorrhagia 6 (7.6%), metrorrhagia 2 (2.5%), hypomenorrhea 1 (1.3%) (Figure 1).

The minimum age of the participant in the study is 14 years and the maximum age is 50 years with a mean age of 31 years (Table 2).

**Table 2:** Age-wise distribution.

Age in years	n (%)
14-19	6 (7.6)
20-25	23 (29.11)
26-30	11 (13.9)
31-35	11 (13.9)
36-40	9 (11.4)
41-45	18 (22.78)
45-50	1 (1.26)
Total patients	79 (100)

Out of 79 patients, 1 (20.3%) was unmarried and 63 (79.74%) were married. Among, the married female most of them had parity of 2 (27.84%).

Among hypothyroid patients, the most common complaint was menorrhagia 5 (45.45%), followed by oligomenorrhoea 4 (36.36%), menometrorrhagia 1 (9.1%), and polymenorrhoea 1 (9.1%). The patient with hyperthyroidism had menorrhagia (Table 3).

**Table 3:** Distribution of different abnormal uterine bleeding patterns among the study population in relation to the thyroid abnormality.

Bleeding pattern	Hypothyroidism n (%)	Hyperthyroidism n (%)
Menorrhagia	5 (45.45)	1 (100)
Oligomenorrhoea	4 (36.36)	0
Menometrorrhagia	1 (9.1)	0
Polymenorrhoea	1 (9.1)	0
Metrorrhagia	0	0
Hypomenorrhoea	0	0

## DISCUSSION

In our study, the majority of the patients were in the age group 20-25 years (29.11%) followed by 26-30 years (1 3.9%). This is quite similar to the study carried out by Pilli et al. which had 58% cases in the age group 21-40 years.^11^ In a study done by Narula et al. about 32.8% of patients belonged to the age group 31-40 years.^12^ In the study by Sangeeta Pahwa et al. about 42% of cases belonged to this age group.^13^ In contrast, a study carried out by Byna P et al. reported that 67.2% of women with abnormal uterine bleeding are in the age group between 35-45 years. About 25.4% of women were between age groups of 46-50 years,^14^ and 55.26% in the study carried out by Kumar AHS et al.^15^

In this study, the most common complaint was menorrhagia which was present in 40.5% of cases. This result is quite similar to that of Moghal et al.^16^ which was about 41 % and quite near to that of the studies of Pilli et al.^11^ about 34% and Sangeeta Pahwaet al.^13^ about 50% and Javed Ali et al. about 42%. The second most complaint was polymenorrhoea in 23 (29.11%) patients which are followed by oligomenorrhoea in 12 (15.1%) patients. This study is similar to a study carried out by Kaur et al.^17^ and Singh P et al.^18^ in which polymenorrhoea was second most complain accounting for 37.5% cases. Fakhar et al.^19^ observed menorrhagia in 45% followed by polymenorrhagia in 30% cases.

The majority of the cases had a parity of 2 (27.84%), about 18.98% were nulliparous of which 16 (20%) were unmarried. This is similar to a study carried out by Gowri M et al.^20^ in which the majority of the cases were parity 2 (39%) followed by nulliparous 20% and primipara 17.6%. In the study carried out by Javed Ali et al.^21^ majority of the cases had a parity of >2 (43%). Forty percentage were nulliparous of which 29% were unmarried. Similarly, in the study carried out by Kumar AHS al. most of them were para 2 (40%) and nulliparous 43%.^22^ In the study carried out by Singh Pet almost all of them were parity 3 (36%) followed by parity 2 (34%).

In the present study out of 79 cases, only 1 2 of abnormal uterine bleeding cases had thyroid dysfunction which accounts for 15.1% cases out of which 11 (13.9%) had hypothyroidism and 1 (1.3%) had hyperthyroidism. Among hypothyroid cases 7 (8.8%) had subclinical and 4 (5.06%) had overt hypothyroidism. This study is similar to study carried out by Kumar AHS et al.^22^ in which out of 200 cases 162 (81 %) cases were euthyroid, 38 (19%) cases had thyroid dysfunction out of which 33 (16.5%) were hypothyroid and 5 (2.5%) were hyperthyroid. Among hypothyroid 21 cases (10.5%) were subclinical and 12 (6%) has overt hypothyroidism. The most common type of abnormal uterine bleeding in this study was also menorrhagia. In another study done by Gowri M et al.^21^ out of 170 cases, 132 (77.6%) cases were euthyroid, 30 (17.6%) of cases had hypothyroidism and 8 (4.7%) had hyperthyroidism. The most common bleeding disorder in this study was oligomenorrhoea followed by menorrhagia and hypomenorrhea. In another study done by Singh Pet al.^18^out of 400 cases, 65% were euthyroid, 26% had hypothyroid, and 9% had hyperthyroidism. The most common type of abnormal uterine bleeding in this study was also menorrhagia followed by polymenorrhoea. In the study carried out by Kattel et al.^23^ thyroid dysfunction was present in 20% of abnormal uterine bleeding cases out of which 19% had hypothyroidism and 1% had hyperthyroidism. The most common type of abnormal uterine bleeding in this study was menorrhagia followed by metrorrhagia. In the study done by Komathi R et al.^24^about 30% of abnormal uterine bleeding had thyroid dysfunction out of which 27% had hypothyroid and 3% had hyperthyroidism. The most common type of abnormal uterine bleeding in our study was also menorrhagia.

Since our study was conducted in a tertiary care hospital, generalizations cannot be done.

## CONCLUSIONS

We found most of the women with abnormal uterine bleeding were euthyroid. In our study among hypothyroid patients most common complaint of abnormal uterine bleeding was menorrhagia, followed by oligomenorrhoea, menometrorrhagia, and polymenorrhoea. The patient with hyperthyroidism had menorrhagia.
